# A case report of Vancomycin in the treatment of Q fever endocarditis

**DOI:** 10.3389/fcimb.2024.1323054

**Published:** 2024-03-19

**Authors:** Xuan Wang, Guangmei Zou, Qianli Wang, Jiao Li

**Affiliations:** ^1^ Department of Cardiac Surgical Intensive Care Unit, Yantai Yuhuangding Hosptial, Qingdao University Affiliated Hospital, Yantai, Shandong, China; ^2^ Department of Cardiology, Tianjin Union Medical Center, Nankai University Affiliated Hospital, Tianjin, China

**Keywords:** Q fever, infective endocarditis, vancomycin, liver injury, *Coxiella burnetii*

## Abstract

The patient, a 43-year-old male, was admitted to the hospital with gradually aggravated exertional palpitations and chest tightness over a 2-day period. Upon hospital admission, a cardiac ultrasound revealed aortic valve redundancy, however multiple blood culture investigations came back negative. Blood mNGS was perfected, revealing *Coxiella burnetii*, and the diagnosis of Q fever (query fever) was established. The temperature and inflammatory indices of the patient were all normal with the treatment of vancomycin before cardiac surgery. But for the potential liver damage of and the *Coxiella burnetii* was still positive in the anti-phase II IgG titer, the doxycycline and hydroxychloroquine instead of vancomycin were applied for the patient. Despite receiving standardized anti-infective therapy of doxycycline combined with hydroxychloroquine, this patient had fever and increased leukocytes following surgery. After the addition of vancomycin as an anti-infective treatment, the temperature and leukocytes improved quickly. During the treatment of vancomycin, a discovery of liver injury may have resulted. These findings provide new therapy options for future professionals.

## Case presentation

The patient, a 43-year-old male, was admitted to the hospital with “exertional palpitations and chest tightness for 2 days”. 2 days ago, the patient experienced palpitations, shortness of breath, and weakness after vigorous activity, which were gradually eased after rest. The aforesaid symptoms increased gradually, with chest tightness without obvious cause. A day ago, he developed a fever with a maximum body temperature of 37.9°C, accompanied by generalized weakness. Other than the aforementioned reported symptoms, he stated to be physically well previously. A systolic murmur of grade II/6 was heard in the left intercostal area of the sternum. Hematology: Leukocytes 11.37×10^9/L, neutrophil percentage 66.5%, platelets 273×10^9/L, hemoglobin 146g/L. Transesophageal cardiac color Doppler examination revealed that the aortic valve had bilobar malformation with severe stenosis and there was strong echogenicity at the aortic valve root, with a size of roughly 15.2*8.1 mm, not excluding an inflammatory mass. EF 71%.

## Treatment and efficacy

After admission, the patient was given ceftriaxone (2g q12h) as an anti-infective treatment. Facial erythema and redness emerged after one day of application. On day 4 of hospitalization, vancomycin, another anti-infective treatment was administered at 1g q12h. Vancomycin valley concentration did not approach the standard after three days of continuous treatment, and the patient was still hypothermic at 37.3°C. Vancomycin was then increased to 2g q12h on day 8 of hospitalization. The patient’s body temperature improved significantly and his white blood cell counts progressively returned to normal. Several blood cultures and sputum cultures were negative during this time period. *Coxiella burnetii* bacteria was found in the blood macrogenome second generation sequencing (mNGS) (high sensitivity whole blood test) on day 8 (sequence number 134, positive reference range 1, the result was reported on day 10). The diagnosis of Q fever (query fever) induced infective endocarditis was considered certain after receiving this new information. During the administration of vancomycin, the valley concentration was monitored and found to be within the normal range. Body temperature, leukocytes, and inflammatory indices were all normal, but liver enzymes gradually increased, with ALT reaching 201 U/L. The effects of liver-protective medications proved to be ineffective. The increase of liver enzymes was suspected to be primarily related to the application of vancomycin. On day 31, vancomycin was reduced to 1g q12h for anti-infective treatment, and liver enzyme levels gradually decreased. On day 38, for that the *Coxiella burnetii* was still positive (14.44U/ml, positive range > 11U/ml) in the anti-phase II IgG titer (inspection on day 34, result on day 37), vancomycin was withdrawn, and a course of 18 months of doxycycline 0.1g q12h combined with hydroxychloroquine 0.2g q8h as an anti-infective treatment was proposed.

The patient’s temperature was largely regulated, and his cardiac function was more stable, so on day 42, he underwent aortic valve mechanical valve replacement and ascending aortic replacement (Wheat’s surgery). Intraoperative aortic valve tissue specimens were taken for mNGS (DNA pathogen detection) and were positive for *Coxiella burnetii* (sequence number 214080, positive reference range ≥1). The patient was extubated on the second day after the operation and continued to receive doxycycline in combination with hydroxychloroquine at the same dose as before the operation. However, the patient developed a fever, with a postoperative temperature of 37.8°C and a maximum temperature of 38.8°C over the next two days. Meanwhile, the leukocyte counts gradually climbed to a peak of 16.47×10^9/L. Vancomycin (1g q12h) was reintroduced to the anti-infective treatment regimen on day 44. The patient’s temperature, leukocytes, and inflammatory indexes decreased gradually the following day. Vancomycin was withdrawn on day 52, while the doxycycline and hydroxychloroquine were continued at the same dosage as before. Following that, a repeat cardiac ultrasound examination revealed good valve and heart function. The patient was successfully discharged with 22 months of oral hydroxychloroquine 0.2g tid and doxycycline 0.1g bid. He has been monitored since discharge and has had no fevers or other symptoms so far. The specific drug adjustments were sort out as **Table 1**.

**Table 1:** Treatment and adjustment reason of the patient.

Date	Treatment	Adjustment reason	
Day 1	ceftriaxone 2g q12h		
Day 4	vancomycin 1g q12h	Facial erythema and redness	
Day 8	vancomycin 2g q12h	Fever and vancomycin valley concentration did not approach the standard	From day 8 to day 31, the temperature, leukocytes, and inflammatory indices were normal
Day 31	vancomycin 1g q12h	Liver damage	
Day 38	doxycycline 0.1g q12h, hydroxychloroquine 0.2g q8h	Coxiella burnetii was still positive	
Day 42	Cardiac surgery		
Day 44	vancomycin 1g q12h, doxycycline 0.1g q12h, hydroxychloroquine 0.2g q8h	Fever	
Day 52	doxycycline 0.1g q12h, hydroxychloroquine 0.2g q8h	Temperature was normal for several days	

## Discussion

Q fever (query fever), is an acute spontaneous epidemic disease caused by *Coxiella burnetii*, which is also known as Q fever *rickettsia*. Sheep, farm horses, and slaughterhouses are the most common sources of infection. It has been found that pathogenic microbes can infect populations up to 18 kilometers away (Agger and Paul, 2014; España et al., 2020). Upon follow up of the patient’s medical history, it was discovered that his hometown neighbors kept animals such as sheep, and the patient may have been exposed to livestock. In humans, the disease is primarily transmitted through the respiratory tract, as well as through touch and the gastrointestinal tract. Clinical symptoms include rapid onset of sickness, fever, malaise, and headache. If the heart is involved, pericarditis, myocarditis, and infective endocarditis may develop, and the valves may be invaded, resulting in the formation of a superfluous organism (Hackert et al., 2020; Steffen et al., 2020; Ghanem-Zoubi et al., 2021). In general, Q fever endocarditis should be considered when there is endocarditis with numerous negative blood cultures, as well as hyperbilirubinemia, hepatomegaly, and thrombocytopenia. Acute Q fever can be diagnosed if the anti-phase II IgG titer is ≥200 and the IgM titer is ≥50 (Fournier et al., 1998). Other diagnostic methods, such as PCR, tissue culture, and mNGS, can also be used to identify it (Kondo et al., 2019; Ji et al., 2021). Doxycycline 0.1g bid is the preferred treatment option for acute Q fever, and fluoroquinolones and sulfamethoxazole are also available for a 14-day course. Doxycycline 0.1g bid coupled with hydroxychloroquine 0.2g tid is recommended for chronic Q fever for at least 18 months, particularly Q fever endocarditis (Patil and Regunath, 2022; Jama et al., 2023). A recent case report showed that azithromycin can treat myocarditis in chronic Q fever infection (Steffen et al., 2020).

According to the 2015 European Society of Cardiology guideline for the therapy of infective endocarditis (Habib et al., 2016), infective endocarditis pathogens are mostly Gram-positive bacteria, and *methicillin-resistant Staphylococcus aureus* (MRSA) treatment should be prioritized. So vancomycin was applied to anti-infective treatment, which is commonly used to treat Gram-positive cocci, particularly MRSA infections (Bruniera et al., 2015). It has been documented that vancomycin can change the cell membrane permeability, infiltrate into the cell (Wu, 2023), and affect intracellular *Staphylococcus aureus* (Li, 2023). Moreover, vancomycin was reported to has effect on *rickettsia* (Barkhatova et al., 1984). Severe human monocytic ehrlichiosis can be treated by vancomycin and doxycycline (Geier et al., 2016). A recent article reported that vancomycin can be used to treat chronic Q fever and high medicinal antibiotics are used to control the spread of infective endocarditis (Kamde and Anjankar, 2022). The temperature, leukocytes, and inflammatory indices of the patient were all normal with the treatment of 2g q12h of vancomycin before surgery. And the result in the anti-phase II IgG titer decreased to be normal (14.44U/ml, positive range > 11U/ml). So we speculate that vancomycin might be effective against *Coxiella burnetii*.

In intraoperative aortic valve tissue mNGS, the patient had a larger amount of *Coxiella burnetii* sequences, indicating that the microorganism primarily penetrated the aortic valve. However, despite receiving standardized anti-infective therapy of doxycycline combined with hydroxychloroquine, this patient had fever and increased leukocytes following surgery. After a department-wide discussion, the temperature and leukocytes improved quickly following the empirical addition of vancomycin anti-infective treatment. There may be susceptibility to infection of the surgical incision or soft tissue, although there are no symptoms and negative tissue bacterial culture. The combination of vancomycin achieved good prognosis, probably for the effect on *Coxiella burnetiid* or the infection of the surgical incision or soft tissue. At present, there is no literature clearly reporting the synergy between vancomycin with doxycycline or hydroxychloroquine. But hydroxychloroquine can increase the intracellular PH, thus affecting cell membrane permeability, which allowing vancomycin to enter the cells and take effect. To sum up, vancomycin may have a perioperative adjuvant effect in the treatment of Q fever endocarditis. There is few previous instance of Q fever treated with vancomycin in the literature (Nielsen and Nørskov-Lauritsen, 2019), and our case may present a novel approach for Q fever treatment.

It is well understood that vancomycin primarily affects renal function. However, this patient experienced liver function decline while receiving vancomycin, making hepatoprotective medications ineffective. The liver enzyme level dropped when the vancomycin dose was reduced, while other factors remained unchanged. We suspect that vancomycin may have impacted his liver function, which was rarely observed in the previous literature (Cadle et al., 2006; Qin et al., 2013). It is suggested that when using vancomycin, clinicians may want to monitor liver and kidney function and evaluate the risk of vancomycin-induced liver injury.

## Conclusion

The mNGS test can be used to diagnose Q fever endocarditis. Vancomycin may have an adjuvant effect in the treatment of Q fever endocarditis. We recommend monitoring the liver function after using high-dose vancomycin anti-infection medication. These provide new therapy options for future professionals.

## Data availability statement

The original contributions presented in the study are publicly available. This data can be found here: [https://www.ncbi.nlm.nih.gov/bioproject, PRJNA1042839].

## Ethics statement

Written informed consent was obtained from the individual(s) for the publication of any potentially identifiable images or data included in this article. Written informed consent was obtained from the participant/patient(s) for the publication of this case report.

## Author contributions

XW: Formal analysis, Project administration, Writing – original draft. GZ: Project administration, Resources, Writing – review & editing. QW: Conceptualization, Investigation, Writing – original draft. JL: Writing – review & editing.
